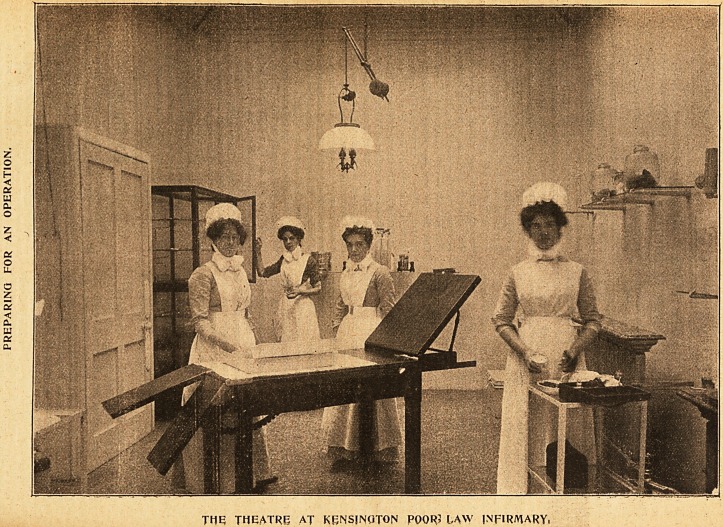# The Hospital. Nursing Section

**Published:** 1907-02-02

**Authors:** 


### The Hospital.

### Nursing Section.

Contributions for "THE HOSPITAL," should be addressed to the EDITOR, "THE HOSPITAL" NURSING SECTION, 28 & 29 Southampton Street, Strand, London, W.C.

No. 1,064.—VOL. XLI. SATURDAY, FEBRUARY 2, 1907.

### Notes on News from the Nursing World.

### THE PROPOSED EXAMINATION OF QUEEN'S NURSES.

THERE has not been any conference between the authorities of the Queen's Jubilee Institute and the superintendents respecting a scheme for the examination of district nurses after their six months' training. Such a scheme is under consideration, but until it has been finally approved by the authorities, it might be misleading to give any details, and it would certainly be premature to criticise. It is probable, however, that at an early date the conditions of admission to the roll will include ability to pass a written examination on a number of important practical questions.

### THE CENTRAL MIDWIVES BOARD AND THE PRIVY COUNCIL.

A SPECIAL meeting of the Central Midwives Board was held on Thursday last week in order to receive and consider a communication from the Privy Council concerning amendments to the Rules. Our representative attended for the purpose of reporting the proceedings, but at the outset a motion was proposed that the sitting should be held *in camera*, and carried; and it was subsequently signified that "no communication would be made to the Press." The Board have, of course a perfect right to exercise their discretion on the question of admitting reporters to their sittings, and, though we regret that they resolved in this case to deliberate with closed doors, we have no doubt that they had excellent reasons for their decision.

### A HOSPITAL FOR NURSES TO AVOID.

WHEN extra nurses are required at the Tiverton Isolation Hospital, they will not find there adequate or healthy bedrooms. At a meeting of the Board of Management, which was held the other day, the Mayor in the chair, the architect reported that when the wards were full and extra nurses were needed, as was the case during a recent epidemic of diphtheria, the day nurse on such occasions occupied the one available bedroom at night, and the night nurse by day. The statement of this fact elicited from a member of the Board the remark that nurses expect "to have a bedroom to themselves." In reply to a question as to whether the arrangement caused much inconvenience, one of the Board said that, anyhow, "it was keeping the bed warm—rather an advantage in the winter." When it had been ascertained that proper sleeping provision would involve an expenditure of about £300, the suggestion was "humorously" made by the same gentleman who advocated a "warm bed" that the new cart used by the District Council to bring in clothes for disinfection might be employed as a bedroom. Ultimately, the Mayor, having deplored the passing inconvenience, thought that as the epidemic had been "tided over," nothing need now be done. Cubicles are bad enough, but to make a nurse who has been working all day or all night in a ward crowded with diphtheria patients sleep in a small room which has been occupied all the previous night or day, is disgraceful. This is clearly a case for the prompt attention of the Local Government Board.

### AN INADEQUATE STAFF AT COVENTRY INFIRMARY.

THE result of an inquiry by a Committee of the Coventry Guardians into the question of the nursing staff at the Poor-law Infirmary is conclusive. They find that the wards are overcrowded, and that the nurses are much overworked. "There was one night nurse"—we quote the words of one of the Guardians—"for 78 patients and another night nurse for 28 patients"; and the report of the Committee says: "At the present time there is one nurse to each 21 patients." It is proposed to remedy this extremely unsatisfactory condition of affairs by promoting two female attendants on female imbeciles to be assistant nurses, and appointing two new attendants. Not one of the four, it will be observed, is a trained nurse, and we do not think that the Coventry Guradians have at all grasped the significance of their own figures, which clearly prove that with 173 patients in the Infirmary more trained help is urgently demanded.

### RESIGNATION OF MISS RAMSDEN.

AT the meeting of the Marylebone Guardians last week the resignation of Miss Grace A. Ramsden, matron of the Infirmary, was announced. This step which, in common with the Guardians, we much regret, is due to failing eyesight. About five years ago Miss Ramsden underwent an operation on one eye, and in the opinion of an eminent ophthalmic surgeon, the other eye has suffered from the amount of clerical work which she has been doing for a long period. In these circumstances, the Guardians have decided, subject to the sanction of the Local Government Board, to add three years to the time of her service for superannuation purposes, and to grant her a superannuation allowance of £67 per annum. Miss Ramsden, who is extremely sorry to relinquish her work, has been at St. Marylebone Infirmary for nearly seventeen years. She entered as assistant matron, and at the end of nearly ten years succeeded Miss Vincent as matron. St. Marylebone Infirmary, thanks largely to Miss Vincent, the first matron, enjoys a unique reputation, and was a recognised training school in days when the standard of Poor-law training was very low. The school was started in connection with St. Thomas's Hospital, and the Nightingale Home at North Kensington was the first nurses' home built in connection with a workhouse infirmary. Under Miss Ramsden's auspices, the high standard adopted when the school was gaining ground has been steadily maintained. She herself was trained at the Edinburgh Royal Infirmary and was afterwards sister at the Liverpool Northern Hospital and night superintendent at the Derbyshire Royal Infirmary. Before her appointment sisters were usually introduced from outside, but Miss Ramsden, having found the experiment of choosing one of the St. Marylebone Infirmary nurses highly successful, has since made it a point of selecting sisters on the same principle. During her matronship an up-to-date operating theatre has been added, and is a great benefit and help in training. The probationers have to take their turn in waiting upon, and helping at all operations, which are very numerous and varied. In recent years the staff has also been increased, and there is now a night nurse and a night probationer on every flat in the building. When Miss Ramsden leaves the Infirmary this month, every one will be sorry, and the nursing staff most of all.

### THE COOK BATHING PATIENTS.

At the last meeting of the Torrington Guardians allusion was made to a statement that the late nurse at the workhouse had ordered the cook to bathe a patient admitted to the infirmary, and that friction had arisen in consequence. The cook, being called before the Guardians, said that she objected to being ordered by the nurse, but that "immediately the matron asked her to bathe the patient in question she did so, and would continue to do so." The cook did right to obey the instructions of a superior official, but the arrangements for the sick must be of a promiscuous character at Torrington, where the duty of washing the patients becomes one of the functions of the cook.

### THE ABOLITION OF PREMIUMS AT ADDENBROOKE'S HOSPITAL.

Against the fact that the permanent expenditure at Addenbrooke's Hospital, Cambridge, as shown by the annual report, has been considerably enhanced by the re-arrangements which have been made in the nursing department under the auspices of the present matron, must be set the gain to the efficiency of the staff in consequence of the abandonment of premiums. Since it was decided not to require probationers to pay premiums, and to give them salaries as well as materials for indoor uniform, the matron has had an immense increase of applicants for training. This means that her choice of selection is no longer limited, and that the institution has the choice of the best class of probationers. Moreover, it has now ceased to be necessary to hire special nurses, and in this particular a substantial reduction in expenditure has been secured. In the end the change must be entirely for the benefit of Addenbrooke's Hospital.

### BAD POLICY AT EDMONTON.

The Edmonton Guardians have determined to adhere to their decision not to appoint an assistant medical officer, although the Local Government Board have intimated to them that "it is essential that in an institution where there are so many acute cases, as is shown particularly by the rapidly increasing number of operations under anæsthetics, there should be a medical officer, or an assistant, devoting his whole time to the duties of his office." While the new infirmary is being built the Local Government Board do not press that the officer shall be resident on the workhouse premises, so long as he can be quickly summoned when his services were required. But in spite of this concession, and although the chairman, at the meeting last week, stated that the number of operations is now 190 as against 30 per annum three years ago, the Guardians, by a majority of three, persist in taking a course which must seriously militate against the welfare of the patients, and also against the institution attracting the class of probationers most likely to reflect credit on it.

### FEVER TRAINING IN IRELAND.

A controversy has arisen between the Lurgan Guardians and the Irish Local Government Board. The latter insist that a minimum of six months' fever training is essential to nurses who seek to qualify for the post of charge nurse at a fever hospital; while the former maintain that with three years' general training, four months and a half in a fever hospital ought to be accepted as sufficient training. In this opinion the Lurgan Guardians are supported by their medical officer, who, in a letter he has written on the subject, says that fever nursing "is not a speciality," and that "a good medical nurse can nurse fever as well as other diseases." There is some force in his contention, but it is now generally admitted that fever training is necessary to nurses who desire to hold a responsible post in an isolation hospital; and therefore we think that the period of six months which the Irish Local Government Board desire to enforce is reasonable.

### POOR-LAW NURSING IN SCOTLAND.

The Scottish Local Government Board have issued a memorandum for the guidance of medical officers of poorhouses in which probationers are trained, that should be studied by the authorities of the English Board at Whitehall. Its great recommendations are that it insists upon an examination of every applicant for the post of probationer by a special officer employed by the Board for that purpose; and that it stipulates for a course of three years' training on the lines of the best general hospitals for every probationer admitted. The Board will continue on their register the names of those nurses who qualified in the past by two years of training in a hospital in which there is a resident medical officer, and these will not be affected by the examinations of new probationers held at the expiration of three years' training. But the valued parchment certificates will only be granted to those nurses who pass this examination; and it is significantly added that "it is expected that all nurses in parochial service will endeavour to obtain this certificate."

### TRAINED MIDWIVES AND THE RATE OF MORTALITY.

In the report of the Sussex County Nursing Association there are many figures which tend to indicate that admirable progress is being made, and there is one fact mentioned which cannot be too widely known. The rate of mortality of infants under 12 months old is lower in Sussex than in any other country in England, and this fact the medical officer of health for the Eastern division attributes to the large proportion of trained midwives—a proportion which is due, he maintains, to the influence of the County Association. It may be hoped that similar beneficent influence will be exercised, with equally good results, by other county nursing organisations.

### THE NEW HOSPITAL AT DUSSELDORF.

In the new hospital which is being erected at Dusseldorf the most modern system of training will be provided for the probationers, and with the medical school and laboratories attached, the would-be German nurse will here find opportunities which have not before been available in the Fatherland for thoroughly learning her work. The hospital itself will contain about a thousand beds, and will include blocks devoted to special diseases, thus allowing for a very detailed system of specialisation, which is always important to nurses. The main administrative block is an imposing stone building, containing quarters for both the medical and nursing staff, separate floors being assigned to each. The whole institution will cover an area of 35 acres, and is enclosed in spacious grounds which will be elaborately laid out, fine shady walks and sparkling fountains being included in the scheme. Every building is to be surrounded by trees and shrubs, and the administrative block will have its own special garden.

### FIRE AT AN ISOLATION HOSPITAL.

An outbreak of fire on Saturday night at the Enfield and Edmonton Joint Isolation Hospital, Winchmore Hill, served to show the presence of mind of both matron and nurse. There were twenty little occupants of two wards in the temporary children's block, ranging in age from three months to eight years—scarlet fever convalescents, who were being looked after by two night nurses. One of the latter, upon going into the bathroom in a corner of the building discovered that it was ablaze. She promptly closed the door, so that the draught should not fan the flames, and also in order to prevent unnecessary alarm in the ward. Then she threw up the window and blew the fire whistle. Having thus summoned assistance, she proceeded to remove the patients to another block, in which work she was speedily assisted by the other nurses, whilst the matron, who on hearing the fire whistle rushed from her bedroom, fixing the hose and the hydrant, played on the burning building until the arrival of the fire brigade. The ward where the fire originated was completely destroyed, but, thanks to the efforts of the staff, male as well as female, no one received any injury.

### THE JUBILEE MEETING OF A FAMOUS INSTITUTION.

The Bishop of London will speak at the Jubilee Meeting of the London Biblewomen and Nurses' Mission, which is to take place in the Caxton Hall, Westminster, on Tuesday, February 19, at 3 P.M. The other speakers will include Professor Clifford Allbutt and Canon Walpole, as well as the Earl of Harrowby who is to preside. The excellent work done by the large staff of nurses attached to the Mission is well known to our readers, who are also aware that a high standard of nursing is maintained. We have no doubt that the proceedings at the Jubilee meeting will demonstrate the fact that the movement was never better appreciated than it is to-day, because, unlike some organisations, it has kept pace with the times, it has been admirably managed, and there has always been associated with its workers the spirit of enthusiasm which does so much to keep alive the interest of the community.

### A START AT QUETTA.

The Quetta branch of the new Indian Nursing Association was opened last month with two nurses, and thanks, in a great measure, to the efforts of the wife of the officer commanding the Royal Engineers, every possible provision has been made for the comfort and convenience of the staff in the Nurses' Home.

### THE NURSES' CO-OPERATION.

The ordinary general meeting of the members of the Nurses' Co-operation will be held at 8 New Cavendish Street on Tuesday next at 5.30 P.M. The business will include the election to two vacancies on the Committee of Management.

### DANCES AND DISTRICT NURSING.

A fancy-dress dance at the Town Hall, Woking, last month on behalf of the Woking and District Benefit Nursing Association realised £55 15s., all expenses being paid by the Committee of ladies who organised the dance. This is satisfactory enough, but at Cardiff, where a ball was recently held on behalf of the Nurses' Institute, the very large sum of £414 16s. 6d. has been handed over to the authorities of that organisation. No fewer than 616 persons paid for tickets for the entertainment, and but for a heavy charge for the City Hall, the proceeds would have been nearly £450.

### SHORT ITEMS.

An institution has lately been opened at Tunbridge Wells for the study of child life. The full course covers a year, and consists of lectures on physiology, hygiene, nursing, first-aid, child nature, and kindred subjects, together with practical work in the nursery, kitchen, and laundry.—Miss M. Barnett has resigned her post as charge nurse at Sculcoates Union Infirmary in consequence of her marriage.

### The Nursing Outlook.

"From magnanimity, all fears above;
From nobler recompense, above applause,
Which owes to man's short outlook all its charm."

### MIDWIFERY IN NEW YORK.

In the year 1905, the number of births attended by midwives in New York numbered 43,834; or 42 per cent. of the total births in the city and its suburbs. This shows that the midwife has a strong hold on the great American city, and gives a special interest to Miss Elizabeth Crowell's investigation of the numbers and training of the midwives thus employed. If New York were a purely American city the midwife would be neither such a frequent nor such an important phenomenon. But it is the port of landing, and very often the permanent home, of a large number of foreign immigrants, the women of whom preserve their old-world liking for the attendance of one of their own sex in their hour of pain and danger. Thus it comes about that of the 500 midwives visited by Miss Crowell (who is assistant secretary of the New York branch of the Public Health Defence League) the great majority were of foreign birth. Austria-Hungary was responsible for 27 per cent.; Italy for 25 per cent.; Germany for 22 per cent., while only 4 per cent. were born in the United States, and the remaining 8 per cent. represented England, Ireland, Scotland, France, Sweden, Switzerland, Syria, Turkey, Holland, Belgium, Denmark, Buenos Ayres, and the West Indies—truly a cosmopolitan collection!

Strange to say Miss Crowell gives the preference among the midwives she has visited to those who have had foreign diplomas. It would hardly be possible to speak in more scathing terms of the American midwifery diploma than she does. Out of the five hundred with whom she deals, "forty-three per cent. held diplomas from so-called schools of midwifery in this country—with two exceptions, schools conducted here in New York city—or certificates from physicians who for considerations best known to themselves, have in many instances seen fit to certify to the proficiency of ignorant, incompetent women desiring to practise midwifery." Again:—"The diplomas of these New York schools are utterly worthless as evidence of training or efficiency on the part of the midwife holding them. In some cases I found that they had been granted to women who were unable to read or write, but who had had the price—66 dollars. There are four such schools in this city." Against these American diploma-ed (we will not say trained) midwives may be set a Russian woman, trained and educated, who had attended between four and five thousand cases, whose husband and son were both physicians, and who could boast, and obtain from both willing confirmation of her claim, that, in the matter of obstetrics, she had taught them all they knew. This woman unfortunately is an exception among midwives, and while she gives the palm to foreigners in the matter of training, Miss Crowell says that of all the 500 of whom she speaks, not more than 10 per cent. could be regarded as capable and reliable.

In face of this statement it is rather alarming to hear that three-fifths of the total number were quite ready to deal with abnormal cases, and never thought of calling in a physician until they found themselves entirely unable to cope with the situation. All of them claimed that they used antiseptics, but Miss Crowell shrewdly says that this claim means very little when the midwife is dirty, her bag filthy, and her general appearance suggests that she is ignorant and incompetent. "As for bags and their equipment," says Miss Crowell, "from a professional standpoint by far the greater number would make fit decorations for a chamber of horrors. Rusty scissors, dirty string, a bit of cotton, a few corrosive sublimate tablets, old rags and papers, some ergot and vaseline, a gum (*i.e.* rubber) catheter, wired, were the usual contents." This insanitary *vade mecum* did not always mean that the midwife had no knowledge of better things, but only that she had learned since she came to America that slovenly methods would pass. Miss Crowell says that foreign midwives who brought out only the usual dirty bag for inspection, had often stowed away "a most complete, compact, convenient portable sterilizer, which they had purchased at home and which the law there had compelled them to use." But in America:—"It is not necessary, nobody cares what we use," was the explanation, when any of them was asked why the good methods were neglected.

It is to be expected that such women should be careless about their work. In the important matter of the care of the eyes of newborn children, the majority of the midwives stated that they used borax or boric acid, and a few used the nitrate of silver solution recommended by the Board of Health. Miss Crowell speaks gravely of midwives who are guilty of criminal practices, and she declares that the machinery of the criminal law is utterly ineffectual in bringing women who so abuse their position to punishment. In fact she admits that the class of women who take up the work is low, and says, "that there is a certain stigma attached to the title 'midwife' must be granted." It should be the duty of all women, midwives, nurses, and laity, to get rid of the stigma thus attached to an ancient, honourable, and important profession, by insisting on a high standard of professional skill and personal character.

### The Care and Nursing of the Insane.

By Percy J. Baily, M.B., C.M.Edin., Medical Superintendent of Hanwell Asylum.

### II.—NURSING THE SICK.

(*Continued from page* 233.)

### 3. *The Skin.*

*Ointments.*—The absorbent powers of the skin are very small so long as the cuticle is intact. It is nevertheless possible to introduce some drugs into the blood by this means, especially in babies, whose cuticle is delicate and thin. The method of administration is called inunction, the drug being mixed in a greasy vehicle forming an ointment. The ointment is to be carefully rubbed into the skin, which should previously be well washed with soap and water and dried. The parts that are usually chosen for the inunction are the inner side of the thighs or arms, the sides of the chest, and the abdomen. In newly-born infants it is often sufficient to spread the ointment on a piece of lint and bandage this to the child's body. Mercury and cod-liver oil are the drugs which are commonly administered in this way.

Various kinds of ointment are frequently used for their local effect in the treatment of skin diseases. These should, unless otherwise directed, be applied spread over pieces of lint.

*Lotions* are usually watery or spirituous solutions which are used solely for their local effect. Such lotions are applied by means of pieces of lint, which should be completely covered with a piece of gutta-percha tissue or jaconet to prevent evaporation. The jaconet should overlap the lint all round by about half an inch at least, and should then be covered with a layer of cotton wool and fixed with a bandage. Some lotions are used for their cooling effect, and are called evaporating lotions. These should be applied by means of pieces of muslin, which should be kept constantly wet, and which, since evaporation is now desired, must not, of course, be covered by anything.

*Liniments* are more powerful preparations than lotions, and are often more or less soapy so as to facilitate their application. They are nearly always ordered to be rubbed into the part. Their object is usually to soothe some deep-seated pain.

*Counter-irritants.*—Drugs which have a local irritating effect are sometimes applied to the skin in order to produce dilatation of the superficial blood-vessels. Some of these are sufficiently powerful to cause blisters or even pustules. They relieve congestion of the deeper tissues, and thus may diminish deep-seated pain. Mustard plasters or mustard leaves, tincture or liniment of iodine, cantharides, etc. are examples of counter-irritants.

*Hypodermic Injection.*—By far the quickest and surest way of introducing drugs into the blood is by hypodermic injection. The drug, in solution, is introduced beneath the skin by means of a hollow needle and a syringe. The dose must necessarily be small—usually about five minims or drops. The barrel of the syringe or stem of the piston is marked off so as to enable the amount of the dose to be accurately measured. The syringe and needle must be kept absolutely clean, and it is now possible to obtain these instruments made entirely of metal, so that they may be boiled to render them aseptic. Except when in use, a silver wire should be kept in the lumen of the needle—when the syringe is about to be used rather more of the solution of the drug than is required for the dose is drawn up into the barrel. The silver wire is then removed from the needle, and the latter fixed to the nozzle of the barrel. The syringe, while being held with the needle pointing upwards, should then be gently but firmly tapped with the finger in order to loosen any air bubbles that might cling to the inside of the barrel. The piston is then pushed up until some of the fluid is driven out of the needle and only the required dose remains. A portion of the patient's skin, which should have been previously rendered aseptic in the manner to be subsequently described, is then picked up between the thumb and finger of the left hand, and the point of the needle driven firmly but quietly into the raised fold. The piston is then slowly pushed up until the syringe is emptied, the needle being partially withdrawn during this part of the operation. As soon as the barrel is quite empty the needle is entirely withdrawn, and the point of the finger placed over the spot from which it has come, to prevent the escape of the fluid, which becomes absorbed by the lymphatics in the space of a few seconds. In selecting the spot for the injection, care must be taken to avoid any vein which may be seen beneath the skin—with this reservation it is immaterial where the injection is made. The arms are the most convenient places, but it should be remembered that the marks left by the needle may be permanent, and therefore in women, who might be likely to wear short sleeves, some other part, such as the back or the outer side of the thigh, should be chosen.

This method of medication is only to be used when the drug is required to take rapid effect to relieve pain or to induce sleep or to soothe the last hours of the patient when he is becoming exhausted in various incurable and painful conditions, such as cancer. In asylums this operation is only rarely entrusted to the nurse, but she should be familiar with it so that she may in an emergency be capable of carrying it out. Morphia is the drug that is most commonly used by this method, but there are many others which it may be convenient to administer in this way.

### *The Bowel.*

The bowel is often made the recipient not only of various medicinal agents, but also of nutriment. Such preparations may be either solid or liquid. When solid they are termed suppositories; when liquid, enemas. A suppository is a small cone-shaped solid body usually about $\frac{3}{4}$ inch long, which is made of a vegetable fat called cocoa butter. This at the ordinary temperature of the air is hard and easily retains its shape, but melts at the normal temperature of the body. The drug—most commonly morphia—which it is intended to introduce into the bowel is incorporated with the cocoa butter.

When a suppository is about to be administered the patient should lie upon his left side, with the knees drawn up and the buttocks near the edge of the bed. The suppository is then dipped in oil and is pushed, point first, through the anal orifice by the nurse. It must be followed up by the finger until it is well beyond the muscular fibres of the anal sphincter to ensure its being retained in the rectum. When the lower bowel is in an irritable state, as in cases of dysentery, etc., there is a tendency for the suppository to be ejected before it has time to melt. When this is the case a warmed towel should be placed over the anal orifice and perineum of the patient, and held there for two or three minutes, by which time this tendency will have been overcome.

(*To be continued.*)

### The Nurses' Clinic.

### THE CLOTHING OF HOSPITAL PATIENTS.

WE all know the clothing in which the average patient is admitted to the ward—the countless petticoats of thinnest possible flannelette in the case of women and children, all fastened with pins instead of buttons; the grimy chemise and drawers; the greasy, broken stays; the much-worn dress; while the men usually have a dirty vest and pair of cotton pants, a more or less patched shirt, and sometimes a belt, as well as coat and trousers. We know these garments well, and treat them with all possible despatch, relegating them to the pigeon-hole, the patient's friends, or the disinfecting-oven, according to their degree of unwholesomeness.

The women also love wearing shawls, and will often resort to much artifice in order to retain one in their possession. These again are abhorred, and rightly so by the nurse, who summarily disposes of them, regardless of discontented looks and murmurs about "catchin' one's death o' cold."

To provide clothing for such a patient to her own satisfaction as well as the nurse's, more tact is needed than is always displayed, and not only tact, but kind common sense and knowledge of what is necessary for the particular case under her care. To the modern hospital nurse, educated to love fresh air, who wears suitable clothing, and is healthy and well fed, open windows are an exhilaration and a vital necessity, and she sometimes fails to recognise how the poor, diseased, ill-nourished specimen of humanity, shivering in her clean bed after the initial bath, abhors a draught as nature abhors a vacuum, and worships that filthy "chest-protector," vest, or shawl as a fetish to shield her from the dreaded bronchitis or pneumonia. "A poor thing but my own" is the thought that finds expression in the clinging regard shown towards these inadequate garments, so, in replacing them by others, a nurse should be careful to supply some equally warm and quite as comfortable, remembering that a thin cotton or greatly worn flannelette night-dress or shirt cannot really feel the same as that old vest, dirty though it might have been.

Badly fed people are more sensitive to cold than those whose diet is always liberal, so that it is worth while to show some consideration for the complaint of chilliness, and not dismiss it with a careless "Oh, you will soon get warm; it isn't really a bit cold to-day."

A vest taken off a patient should always be replaced by another, and if the night-dress is thin add a warm bed-jacket and put an old blanket next the patient under the top sheet for a few hours, also a hot bottle to his feet. If a nightgown which is open at the back has to be worn for some time, owing to an abdominal operation or for any other reason, a nurse sometimes forgets to change it for one of the ordinary pattern when the patient begins to sit up in bed, with the result that a considerable portion of the back is left without covering, thus affording ample opportunity for a dangerous chill. The same thing may happen when a patient is turned over on her side, if care is not taken to put a pillow at the back and to tuck a blanket between it and the shoulders.

Unless it is very hot summer weather, a jacket or cape should be worn when sitting up in bed; it is a good plan to have a separate one for the day, changing it at night for one of thinner material.

With convalescents who are able to get about the ward, the mistake is often made of allowing them to go too thinly clad. In the case of men this is particularly so. The first time they are got up on to the couch for half an hour they are often loaded with blankets and rugs, so that they can scarcely move a limb; but as soon as they can walk about they are allowed to do so wearing only a ward dressing-gown over their shirt and a pair of slippers. I have even seen the socks or stockings omitted. As men cannot supplement their attire by petticoats as women patients do, it is most necessary that they should wear trousers or pants whenever they are able to walk about, and it is absolutely essential that the feet and legs should be warmly and adequately covered. Many an unexplained relapse during convalescence is undoubtedly due to a chill contracted in this manner, especially among children or lads too young or too thoughtless to complain of feeling cold.

If the patient is placed in an easy chair for the first time of getting up, a rug should be spread over the seat in such a manner that it will reach quite to his heels, so that it can be easily wrapped round his knees as he sits down, and preclude any possibility of a draught chilling the back of his legs. The chair, too, should not be in a direct current of air, nor face too strong a light. If on a ward balcony it ought not to be just opposite the door leading into the ward, or a draught will be felt. The eyes must be protected from the sun by a light shady hat or an umbrella. If in a ward the chair should not be too near the fire. Warm list or felt slippers are best for ward wear, with woollen socks or stockings. There ought to be also a good supply of woollen or flannel vests for every ward, and if not provided by the hospital an energetic sister or nurse can generally interest her own friends in her work sufficiently to get them made for her, doubtless as a free gift.

Where there are grounds attached to the hospital, patients should be made to put on boots before going out to walk, in case of getting damp feet, also for economic reasons connected with the ward slippers.

### To Nurses.

WE invite contributions from any of our readers, and shall be glad to pay for "Notes on News from the Nursing World," "Incidents in a Nurse's Life," or for articles describing nursing experiences at home or abroad dealing with any nursing question from an original point of view, according to length. The minimum payment is 5s. Contributions on topical subjects are specially welcome. Notices of appointments, letters, entertainments, presentations, and deaths are not paid for, but we are always glad to receive them. All rejected manuscripts are returned in due course, and all payments for manuscripts used are made as early as possible after the beginning of each quarter.

### Illustrations of the Life of a Modern Nurse.

THE THEATRE AT KENSINGTON POOR LAW INFIRMARY.

PREPARING FOR AN OPERATION.

### Incidents in a Nurse's Life.

### MY FIRST NIGHT IN A CHILDREN'S HOSPITAL.

I commenced training in a children's hospital, and in due course was put on night duty. I had charge of the girls' ward, with fifteen patients, ranging from 10 weeks to 12 years old, each more or less interesting in her own way.

As the lights were to be kept low in the ward, I found myself installed in a little day-room adjoining, with a basket of socks to mend in the intervals, when the children did not want attention. Rather sleepy work, I thought, for the middle of the night. However, the little patients did not give me the chance of going to sleep. No doubt, too, being my first night on duty, I was over-anxious, and when day came I am afraid the matron did not find the pile of unmended socks much reduced.

I was given a book in which I was to keep a record of what sort of night each of the children passed; especially was I to notice if any of the hip children cried out in their sleep. I had just settled down to my first sock when suddenly, through the silence of the ward, a sharp cry rang out, familiar to all who have nursed children with hip disease. The question was, which child had cried? I threw down my work, and ran into the ward, but, alas! all was still and quiet. This happened once or twice during the night, and I was obliged to confess, with shame, in the morning that I was unable to say which child had cried out. However, these difficulties are overcome by experience, and after the first week I knew directly whose voice it was which so weirdly broke the silence. It was now time to make the poultices and fomentations, which had to be applied, and the foods for those who had to be fed during the night. Three or four, happily, were so far advanced towards convalescence as to sleep fairly peacefully all through the night.

There was one poor little baby of ten weeks who, owing to bad feeding and mal-nutrition, did not look more than a fortnight old. She had to be fed constantly in small quantities, and, being too weak to suck, I had to administer the food with a syringe. This needed some skill, to choose the right moment, and to get the food down between the feeble wails, or I might have choked the poor mite, instead of nourishing her.

After all had been arranged comfortably, and I had had my own early morning meal, I once more settled down to that pile of socks, which laid rather heavily on my conscience.

A quarter-of-an-hour, perhaps, passed away, when, clang, clang, went the front-door bell. "Certainly," I thought, "night duty cannot be called dull work." I knew what that peremptory summons meant—an accident of some sort, and a pretty serious one to be brought in the middle of the night. The nurse in the downstairs ward would, I knew, go to the door, but I listened anxiously. If it were a girl she would come up to my one empty bed. Presently I heard my name called, and ran down, to be told that a little girl was being carried in from a house in the neighbourhood, which was on fire. This news had been brought by one of the ready messengers, always to be found at such times. I ran upstairs again as fast as I could, wondering how much the poor little soul was burnt. First I went to tell matron (she always had to be awakened in case of any emergency, as we nurses were all young), then back to the ward to get all in readiness for the reception of my new patient.

Matron arrived just as the men were carrying the poor little girl upstairs, and we were soon busy, doing all we could for her comfort. We saw at once that she was very badly burned, and the chance of her recovering was small.

As it was now between four and five o'clock matron elected to sit up herself and watch the child rather than call out one of the day nurses, for I should soon have the breakfasts, and early morning work to do, and could not possibly give the poor child enough attention. If she could only be tided over the time of collapse resulting from the shock we might pull her through, matron said. The noise of the new arrival had roused many of the little patients and they all had to be soothed and settled off again.

In a few hours time all the work of the busy early morning hours was done, and I retired, thinking I should not soon forget my first night, and wondering if I should find my poor little new patient still alive when I came on duty again.

### The Consumptive at Home.

### AN ALTERNATIVE TO SANATORIA TREATMENT.

The prevention of tuberculosis by the simple method of carrying the patient away to a spot where he can no longer infect his family, is far easier in London than in rural districts. When the hospital and the convalescent home have done their part, and maybe failed to effect a cure, there is always open to the phthisical patient a last resort from the crowded London lodging in the shape of Poor-law Infirmary, and to an ever-increasing extent this resort is made use of by persons who would otherwise be a source of danger to the community. But "to go into the workhouse" has still in the country its old ominous sound, nor is the reluctance to resort to its hospitality always ill-founded. The following notes of a case recently dealt with may serve to illustrate some of the difficulties which exist with regard to tuberculosis in country districts, together with a suggestion for their remedy.

The patient is a young man not yet twenty years old. He is the third child in a family of eleven children, all of whom except the eldest daughter, in service, inhabit a cottage containing four rooms. The father is in regular work at 25s. a week, and the eldest son is also at work, but the remainder of the family are young children. Reginald was himself employed at some engineering works about two miles from his home when he contracted influenza, neglected it till pneumonia supervened, and was finally taken to the County Hospital, from which he was discharged after many weeks with both lungs seriously affected. With the help of some friends he was sent to the West of England Convalescent Home at Weston-super-Mare, and there remained until the approach of winter. By good fortune a free bed was then secured for him at St. Catherine's Home at Ventnor. And from Ventnor he returned, in late April 1905, almost cured. The medical report stated that the disease was quiescent, and it was hoped that he would soon be fit for some light employment. Meantime, as his home was situated on high ground on a spur of the Chilterns, it was thought that he could do very well there for a time. There was, in fact, nowhere else for him to go. Alas, he returned to sleep in the same bed with his brother, in a tiny room with the smallest possible aperture for a window, and from the first day he began to travel downhill. A room was rented and furnished for him next

THE CONSUMPTIVE AT HOME—*continued.*

door, but the window was again about two feet square, and only half of it was made to open. A summer spent sleeping in this way and wandering restlessly about all day, when he was not sitting in the stuffy kitchen among a crowd of little brothers and sisters, reduced poor Reginald to a deplorable condition. All the skill, money, and care freely expended in the endeavour to restore him, were easily neutralised within a few weeks, and this in air reputed the best to be found in England. A suggestion that Reginald should be accommodated in an open shed in the adjoining field, was indignantly repudiated by his sorrowing relatives. They would resign themselves to the death of their favourite son, if it were the will of God, but to have him lying out in a field, exposed to the damp and dangerous airs of night, was too much to expect. Finally a great effort was made on his behalf, the head of an Open-air Sanatorium within six miles of his home generously promising to receive him for the winter if sufficient money were forthcoming to defray the expense of his food. And so from October till the end of May he lived out of doors in a châlet, just holding his own, with all that the highest skill could do for him. During all this time his relations were allowed to visit him, and slowly, through the zeal of the patient, who never wavered in his allegiance to the system on which he knew his life to depend, they came to understand. When the time came for his return once more to his home, both he and they eagerly adopted the old suggestion that it should be a return to life in the open air, modelled as far as possible on the sanatorium. Once more an effort was made, and this time with some hope of making permanent provision for the sick lad. An excellent site was found in the cottage garden, sloping down towards the south and surrounded with grass land. Here a shed of the roughest description has been erected, with corrugated iron roof lined with felt. It is twelve feet square; on three sides are windows four feet square, made to open top or bottom as required, and on the fourth side is the door. Here, then, the consumptive is happily established, the wonder and admiration of a simple population in the hamlets around, who have learned all their lives through to regard fresh air as the one deadly enemy always at hand to bring them to destruction. The conditions under which he lives are as nearly perfect as could be devised for one in his state, and since his return it is a fact that his health has undergone a striking improvement. The chart of his temperature, which he keeps with religious accuracy, is shown occasionally to nurse and doctor, but there is small need at present for the attendance of either.

Now the cost of the shed was £8, and the cost of the winter spent in the sanatorium was £18. The money spent altogether over this one patient, taking the cost of his hospital treatment and the various homes together, cannot have been much under £70.

Why should it not be possible to organise a system for providing phthisical patients with the means to continue the open-air life they learn to value in sanatoriums after their return home? To attempt to enforce the treatment before the patient has practised it under discipline and learned its life-giving properties can but result in failure. Yet almost every country cottage, however overcrowded, offers facilities for erecting a shelter in its vicinity where the consumptive can get the element he needs, and, what is almost as important, be removed from the danger of infecting others of his family. And when once the lesson has been learned, no reluctance will be shown in practising it.

It is hardly to be supposed that the family will in any case have sufficient initiative to carry out the arrangements unaided. But it might, we think, well be a part of sanatorium treatment to insist on the provision of some means for carrying on the system at home when the patient is discharged.

The chronic consumptive, sowing widespread the seeds of his disease, is to be found in every hamlet in England. To found sufficient homes to hold them all is beyond the utmost stretch of charity. But every patient established in his own home, among his own people, under open-air conditions, is much more than "a hopeful case." He is an object-lesson, to all his numerous visitors, of the benefits of fresh air; and his scorn of draughts, his amusement at the condolences poured upon him, and his scraps of well-remembered lore from the sanatorium, make an even greater impression than all the excellent maxims of the district nurse herself.

### A Case of Nervous Breakdown.

SHE was called Nurse Elizabeth in the nursing home, but to one patient she was Saint Elizabeth from the first day. It is possible that the outward observer might see in her nothing beyond a sensible, earnest-minded, capable woman; for helplessness alone can draw out her strength and boundless sympathy. And even those who know her well would be puzzled to define wherein lay her charm, or why she succeeds where others fail.

Unless it is that beyond her quick perception and true mother's instinct she has a heart that has learnt through suffering and personal loss to sympathise with and understand another's need.

The case she undertook was briefly that of a worn-out selfish, miserable, hopeless woman, full of imaginary diseases, with a mind so perverted as to be almost incapable of even wishing to be herself again. She had lost nearly two stone in weight and was unable to eat or sleep, or to get rid of the idea that she was a hopeless invalid.

Add to all this a pulse that raced feebly around 130, an adoring husband spoiling her at every turn, anxious friends giving in to every absurd whim, add also the opinion of a well-known London specialist: "almost the worst case I have seen yet," and it will be seen that Nurse Elizabeth did not succeed because circumstances were easy.

In the four months' conflict with this ill-balanced brain she aimed from the very first at nothing less than a complete recovery, which, judging entirely from her own observations, she pronounced possible.

This belief she gradually managed to convey to her patient's mind, first by inspiring a longing to be well and then by the force of her own conviction imparting the possibility. Nothing ever appeared to discourage her, through weeks of sleeplessness she still maintained its temporary character.

A hundred times over she must have patiently reasoned through the long nights "Don't toss about from side to side like that—you can lie still if you want to. Now don't try to go to sleep. Just lie still and think of the happiest day in your life. It doesn't matter if you sleep or not." This unimportance was the right line to work on, and the metallic tones of the other nurse repeating at intervals "If you won't go to sleep you will lose your reason directly," soon ceased to haunt or worry, and the conjured-up visions of an asylum gradually melted away from the patient's remembrance. It was the same with food; she did not bring it in with an expression of "Here it is, and I am going to stand here till it is eaten." Carefully she explained the reason of each order, and successfully dealt with every objection, inspiring with each word a growing desire in her patient's heart to consume all available food and ask for more.

She never talked very much, and when she told about outside things it was of the country, the sea, and the moors that she spoke. Or perhaps it would be a story of a little baby she had nursed and loved, but nothing ever that was long or prosy, and, by the time the happy ending of its mother's joy at her child's recovery was reached a dreaded meal would unconsciously have had a satisfactory conclusion too.

Once coming in with a bunch of heather and an amusing experience with a little nursemaid to relate she stopped suddenly.

"Why, what is the matter? Have you developed a new disease since yesterday? It was a tumour then, wasn't it? and appendicitis the day before and pleurisy the day before that? Well, what is it to-day?"

A sigh was the only answer at first, then, after a due amount of pressure—for Nurse Elizabeth believes in finding out the cause before attempting to remove it—she extracted the information that "Matron and nurse had been talking outside the door that morning and nurse had said she was 'tired of such a spoilt woman;' and Matron said 'she would never be any better, so Nurse Elizabeth needn't think so'—I know it was me they meant, and (beginning to weep) I can't live and be like this always.'"

There was a long pause before Nurse Elizabeth began to answer, then quietly and sensibly, she went over the old ground, pointing out improvements, comparing the present with the past, and painting the future with glowing colours, ending up with a smile that took every bit of hardness out of the usual ending: "But you know it depends upon yourself, and you will only get out of this just what you put into it."

It was not once or twice she had to reason so, but daily, sometimes almost hourly. This overheard conversation was possibly imaginary—and Nurse Elizabeth knew it—but it made no difference to her invariable patience. It was real to the invalid and must be dealt with accordingly.

One day stands out from all the others, it happened after the treatment had been continued about a month.

The doctor, who had hitherto been anything but encouraging, suddenly turned to her with a beaming face—"You've done splendidly, nurse," he said, enthusiastically—"splendidly; I only wish I had seven other nurses just like you this moment."

Nurse Elizabeth did not smile at the praise to herself, but kept it for afterwards, when she said to her patient: "You see, I knew you could do it, if you really meant to."

This was the first recognised advance towards recovery, but perhaps the next three months of semi-convalescence were more trying.

She had to put up with days together of unreasonable bad temper and long fits of sulky silence. Of these she would take no apparent notice, though indirectly she was making every effort to help her patient to conquer the demon of depression. Then, when at length cheerfulness was restored, without a reference of any kind to the cross and ungrateful words, she would heap coals of fire upon the offender's head by extra consideration and gentleness. She chose a bright sunny morning when it felt good to be alive to make her few remarks upon giving way to depression. "That is all," she ended up with, "and I don't think now you are so well you will ever do it again." And so through all the ups and downs she remained always the same ever kind and sympathising friend, ever appealing to all that was good in others, never thinking of herself. Truly "a heart at leisure from itself, to sooth and sympathise."

Slowly but surely the end so longed for was reached, and one day a strong, healthy girl might have been seen at Victoria Station, looking longingly as the train moved away, at a face framed in an ordinary blue bonnet, leaning out of the carriage window, as through a mist of tears she saw the last of the woman whom she had grown to regard almost as a saint.

. . . . . . .

Late that night, sitting over the fire, my husband said to me, "So you liked nurse all the time?" "Liked her?" I answered, "I think I shall always love her, for no one could believe how good she has been to me."

"Yes," he said almost reverently; "I think your name for her was the right one. I hope I shall never forget to be grateful to her for bringing me back my little wife just like she used to be."

The rest of what he said does not matter here, as it had no further reference to SAINT ELIZABETH.

### Queen Alexandra's Imperial Military Nursing Service.

POSTINGS AND TRANSFERS.—Miss M. L. Harris, sister in Queen Alexandra's Imperial Military Nursing Service, has been transferred to the Military Hospital, Devonport, from trooping duty, s.s. "Plassy"; Miss E. H. Hordley to the Military Hospital, Portsmouth, from South Africa; Miss K. Pearse to the Military Hospital, Pretoria, from the Military Hospital, Standerton; and Miss A. A. Wilson to the Military Hospital, Middelburg, Cape Colony, from the Military Hospital, Wynberg. Miss E. G. Barrett, staff nurse, has been transferred to the Queen Alexandra Military Hospital, Millbank, London, from the Military Hospital, Portsmouth; Miss K. Roscoe to Egypt, from the Royal Victoria Hospital, Netley; and Miss H. M. E. Macartney to Egypt, from the Royal Herbert Hospital, Woolwich.

### Queen Victoria's Jubilee Institute for Nurses.

THE QUEEN has been graciously pleased to approve the appointment of the following to be "Queen's Nurses," to date January 1, 1907.

ENGLAND AND WALES.—Amelia Annie Alder and Bertha Cobden Hosking, district training at St. Olave's; Margaret Hannah Bevis, Birmingham (Newhall Street); Marie Evelyn Crouch, Blackburn; Grace Addenbrooke, Sarah Ellen Lebart, Fanny Sims, and Florence Mabel Stead, Metropolitan Nursing Association: Emmeline Denby and Isabella Randall, Brighton; Grace Edith Burgess, Camberwell; Margaret Mary Crowe and Mary Stephenson, Cardiff; Katharine Maude Bladen, superintendent at Cheltenham at date of affiliation; Marie Amelia Stuart Edwards, Ada Josephine Godby, Emma Jane Hall, Kate Emma Gertrude Taylor, Emma Anita Mary Walters, Mary Ann Wilkinson, and Elizabeth Williams, working at Cheltenham at date of affiliation; Kate Annie Cooper and Laura Frances Touch, East London (Southern Division); Jennie Duffy, East London (Stepney Division); Ada Powell, Gloucester; Elizabeth Moorhouse Barlow, Hull; Lindora Louise Eskell and Mabel Thorp, Kensington; Florence Amelia Gibbons, Leeds (Holbeck Home); Ruth Oates and Mary Elizabeth Saunders, Liverpool (Central Home); Gwen Lewis-Jones, Liverpool (Derby Lane); Hilda Elsworth and Emily

Richardson, Liverpool (Overton Street); Ada Duckworth, Liverpool (Shaw Street); Hannah Harrison, Liverpool (North Home); Alice Mary Gertrude Ferguson, Manchester (Hulme Home); Mary Jane Halkett, Maud Mary Hughes, and Louisa Gwendoline Ogden, Portsmouth; Mary Evans, St. Helen's; Alice Mary Goodman and Emma Sutton, Salford; Mary Ellen Hughes and Grace Trotter, Shoreditch; Elizabeth Mary Haynes, Southampton; Nora Vincent Stewart, Sunderland.

Scotland.—Jane Douglas, training at Dundee; Margaret Walker Arnot, Elizabeth Fyfe Beck, Flora Cameron, Elizabeth Cox, Elizabeth Davidson, Mary Douglas, Beatrice Mary Harvey, Jessie Maclean, Agnes McGregor, Annie Smith McMillan, Agnes Mercer, and Catherine Cooper Trotter, Scottish District Training Home, Edinburgh.

Ireland.—Gretta Coughlan, Lily Geatens, Ellie Hogan, and Ellen Kennelly, training at St. Lawrence's Home, Dublin; Adelaide McGibbon Campbell, St. Patrick's Home, Dublin.

Transfers and Appointments.—Miss Helen Court has been appointed superintendent of Coventry District Nursing Association; Miss Annie Aldridge to Darlington; Miss Cornelia Bennett to Three Towns; Miss Mary A. Harrod to Highcliffe (temporarily); Miss Maude E. Jacocks to Heanor; Miss Jessie Kennett to Dartmouth; Miss Sarah Parry to Portmadoc; and Miss M. H. Spalding to Pontyclun. Miss N. Lloyd has been transferred to Wykeham from the Harpurhey Home, Manchester; and Miss Florence Moore to Orielton from Pontyclun.

### Everybody's Opinion.

[Correspondence on all subjects is invited, but we cannot in any way be responsible for the opinions expressed by our correspondents. No communication can be entertained if the name and address of the correspondent are not given as a guarantee of good faith, but not necessarily for publication. All correspondents should write on one side of the paper only.]

### A PROBATIONER LOSES HER SIGHT.

Mr. Michael J. Turner, of 3 Comberton Road, Upper Clapton, N.E., writes: On behalf of my daughter, Nurse Helen Turner, I beg, through the medium of your columns, to thank "Anonymous" for the kind gift announced in your issue of January 26, and still more for the very evident and welcome sympathy that prompted the action.

"Nurse S. V. F." writes from Westminster Nurses' Home, 27 Queen Anne's Gate: As a very great friend of the probationer who lost her eye at the West Ham Infirmary I wish to express my feelings of gratitude to the anonymous donor of £5, and to say how greatly the feeling of sympathy and kindness thereby shown is appreciated by the sufferer and her friends. The girl loved the work, and the thoughts of being unfitted to continue in the profession was by far a greater worry to her than the loss of the eye. That she was appreciated by those she worked under is shown by the words of the ward sister under whom she was working. She said to me: "If there were more girls like Nurse T—— in the nursing profession it would be a blessing, as she is the sort of girl wanted." Being strong and healthy and only twenty-three years old, and in a position in which it is absolutely necessary to gain a livelihood, one cannot but hope that she may succeed in some way in continuing in the work. It is uncertain as to whether the sight of the other eye will be satisfactory, as she is still under treatment at Moorfields Eye Hospital.

### MIDWIVES' DEFENCE ASSOCIATION.

Miss Louise M. Lee writes, as secretary *pro. tem.*: Will you allow me, through the medium of your columns, to call the attention of the midwives of London to the formation of a Defence Association by the midwives of the Royal Maternity Charity of London. We hope that other midwives not on the staff of the Charity may be induced to join us, so that the Association may be self-supporting. I shall be pleased to give any information to intending members. Letters may be addressed to the Secretary of the Midwives' Defence Association, 31 Finsbury Square, E.C.

### WORKHOUSE NURSING.

"Desperandum" writes: I was glad to see "Superintendent Nurse's" letter in your paper last week. I can fully endorse all she says; but, unlike her, I have given up the idea that I can ever live at peace in Poor-law institutions. I have held three posts as superintendent nurse. The last was worse than the other two. I had one night nurse for nearly 100 patients, and she was a woman well advanced in years and totally untrained, besides not being able to read or write, or, as I once heard a patient remark, "She can neither tell the time of clock by day or night nor the number on a reel of cotton." She was also intemperate. When medicines had to be administered at night I used to tie different-coloured cotton on the various patients. But I afterwards found that one of the patients was awakened invariably to read the names, as the nurse could not remember. I complained to the master and matron, to the doctor and to the guardians; but no notice was taken. When the Local Government Board inspector visited, he suggested various improvements. These were not carried out, and, when I asked why, I was told by the officials that inspectors were paid to make a fuss, but no one took any notice of what they said, and I should get used to it in time. However, I did not, and after spending nearly ten years of my life in Poor-law work—I was training for nearly five years at a large Poor-law infirmary—I have left it, I hope, for ever.

### Appointments.

[No charge is made for announcements under this head, and we are always glad to receive and publish appointments. The information, to insure accuracy, should be sent from the nurses themselves, and we cannot undertake to correct official announcements which may happen to be inaccurate. It is essential that in all cases the school of training should be given.]

Basingstoke Union.—Miss Charlotte Alice Phipps has been appointed superintendent nurse. She was trained at the Royal Infirmary, Bristol, and has since been nurse at East Preston Union Infirmary and charge nurse at the Western Hospital, Fulham, London.

Birkenhead Union Hospital and Infirmary.—Miss Laura Beatrice Paul and Miss Madelaine Mary Graham have been appointed charge nurses. Miss Paul was trained at Farnham Poor-law Infirmary, and has since been charge nurse at Bolton Union Hospital. Miss Graham was trained at Toxteth Park Union Infirmary.

Broadstone Jubilee Hospital, Glasgow.—Miss Minnie Templeton has been appointed matron. She was trained at the Glasgow Western Infirmary, and has since been sister at Greenock Infirmary.

Fever Hospital, Birkenhead.—Miss Ethel Bentley has been appointed charge nurse. She was trained at Salford Union Infirmary, where she was afterwards sister. She has also been sister at the Heswall Sanatorium, Cheshire.

Holborn Union Workhouse.—Miss Janet Ray has been appointed charge nurse. She was trained at West Ham Infirmary, and has since been charge nurse at the Bridge School, Witham, Essex, and sister at the Lambeth Parish Schools, Elder Road, West Norwood.

Joint Hospital, Brighouse.—Miss E. Simpson has been appointed charge nurse. She was trained at Mill Road Infirmary, Liverpool, and has since been charge nurse at

Walton Infirmary, Liverpool, and charge nurse at the West Derby Union, Belmont Road, Liverpool. She holds the Central Midwives Board certificate.

Normanton and District Joint Isolation Hospital.—Miss Mary O'Brien has been appointed matron. She was trained at Torbay Hospital, Torquay, and has since been charge nurse at Southwark Infirmary, East Dulwich; assistant matron at the City Fever Hospital, Grafton Street, Liverpool; and matron at the Leeds Home of Recovery.

Royal London Ophthalmic Hospital, London.—Miss Mary L. Pollett has been appointed lady superintendent and matron. She was trained at the London Hospital, and has since been sister at Radcliffe Infirmary, Oxford, assistant matron at Nottingham General Hospital, matron at Abingdon Hospital, and matron at the Hospital for Epilepsy and Paralysis, Maida Vale, London.

Suffolk General Hospital.—Miss Marian Robertson has been appointed night sister. She was trained at the Hospital for Children, Edinburgh, and at Charing Cross Hospital, London. She has since been temporary night sister at Charing Cross Hospital; sister at Tunbridge Wells Hospital; and superintendent of a nursing home at Las Palmas. She has also done private nursing.

### Presentations.

Lyndhurst District Nursing Association.—Miss Clutterbuck, who has been district nurse at Lyndhurst, Hants, since December 1894, has received a handsome present, chiefly from the poor in her district, who themselves planned and collected for it, "as a mark of affection and appreciation of her twelve years' love and care." The gift consists of a pianette and a settee, two guineas for music, etc., and a book containing the subscribers' names.

### Mrs. Beeton's Book on Household Management.

The task of reviewing a new edition of this world-famed book, which has come, by its general excellence, to be recognised as an indispensable adjunct to every middle-class household, appeared formidable at first sight. What could be said about this classic of the kitchen that has not been said already? However, a glance into the depths of the new edition quickly altered the point of view and revealed so many new good points in the book that the difficulty will be to find space to give them justice. The chapter devoted to invalid cookery stands out in strong relief. It is very excellent indeed, and includes all the latest recipes for beef-tea, chicken-broth, and soups, with many light delicate ways of cooking fish, poultry and meat. These latter recipes would make very palatable and suitable dishes for those who have to provide dainty little dinners for people with delicate digestions. A chapter on peptonised foods and another on diabetic foods will be found of great use where such foods are required.

The book is stamped as up to date by the very good and simple recipes given for chafing dish and casserole cookery. Such things were unknown in Mrs. Beeton's days. The whole conditions of life have altered. People have larger incomes and more luxurious habits, but a comparison between the tables in the original and the present book gives food for thought! It brings out the fact (realised fully by few women) that money nowadays is only worth about half what it was worth in Mrs. Beeton's time. Rent is higher, servants' wages are double, and a more luxurious scale of living is the custom. Never then was there a time when the careful study of household management was more important. Life in these days is so full, either of work or pleasure, or a combination of both, that girls no longer care to give much time to so commonplace a science as housekeeping, but there is danger in this neglect. What if the meaning of the old word "home," so characteristic of the English nation, gets lost by the ever-increasing custom of living in hotels and dining at restaurants because women will no longer find a pleasure in mastering the difficulties of domestic management, and servants will no longer feel they are part of the family with whom they live, nor take pride in doing work that fits them better than any other kind of occupation could, to be wives and mothers in a comfortable home of their own. Let them ponder over the meaning of the word "lady," and then, perhaps, they will take more trouble to be the "dispenser of the loaf" and the gracious hostess that makes for the peace and happiness of a household.

No one need feel that this most useful book is beyond their reach, for the proprietors of the Lemco Company have arranged with the publishers of Mrs. Beeton's book on household management (price 7s. 6d.) to give a copy free of charge in exchange for weight coupons representing five pounds of Lemco if collected before 31st of March next. All Lemco preparations bear a little round coupon on top of the cork under the capsule, and it is these coupons which are available for the book.

The Lemco Company have always felt that the more they could interest their customers in cooking, the more popular Lemco would become, and following on this policy they have given away millions of original cookery books. Their latest is called Lemco dishes for all seasons, in which chapters are devoted to articles in season each month, a special chapter giving 20 recipes for nourishing sick-room dishes. Anyone can obtain a copy of the Lemco Cookery Book by writing name and address on a Lemco wrapper and forwarding it to the Lemco Company, 4, Lloyds' Avenue, London, E.C. Among the many new and useful features in Mrs. Beeton's book is the chapter devoted to "Vegetarian Cookery." The authoress expressed the thought that it would take centuries to develop the art of preparing vegetable food that would be sufficient for man's subsistence without meat, but the world moves quickly, and vegetarianism becomes daily more popular. This is an age that considers the dietetic qualities of food more than that of any previous time. Very few people nowadays live to eat, and most people realise that in eating to live the question of diet and the nutritive qualities of food is an all-important one, that if carefully considered and acted on will save doctors' bills and keep the mind and body vigorous and healthy, and proof against many of the dangers of disease.

Mrs. Beeton's book in its present form will guide people to take a right course in regard to the question of diet, and will certainly continue to mitigate the difficulties of housekeeping in every household where it finds a place. It deserves even a longer life than its predecessor.

### TRAVEL NOTES AND QUERIES.

By our Travel Correspondent.

Holland in Early Spring (H.E.R.).—Yes, I think I can tell you of accommodation to suit your terms, but I must have more particulars before I can be of real help to you. Tell me exactly how long your holiday will last, and the precise sum you can afford to spend, including the journey. Holland is a small country, and you could see many interesting cities without much travelling. Are you going alone? If so, I should not advise Holland. Write me at once, and give full particulars.

### Notes and Queries.

**REGULATIONS.**

**The Editor is always willing to answer in this column, without any fee, all reasonable questions, as soon as possible.**

**But the following rules must be carefully observed.**

**1. Every communication must be accompanied by the name and address of the writer.**

**2. The question must always bear upon nursing, directly or indirectly.**

**If an answer is required by letter a fee of half-a-crown must be enclosed with the note containing the inquiry.**

*Midwifery.*

(186) Can you tell me of any nursing institute in London where they employ nurses who are only trained in midwifery?—*Colchester.*

Write for advice to the Midwives' Institute, 12 Buckingham Street, Strand, W.C.

*Urine Testing.*

(187) Can you recommend me an inexpensive book on urine testing?—*Norfolk.*

Get Dr. Watson's little book on urine testing, price 1s. 1d. post free, from The Scientific Press, 28 and 29 Southampton Street, Strand, London, W.C., or order from your bookseller.

*Hospitals in Brighton.*

(188) Can you tell me if there are any hospitals in or near Brighton?—*An Anxious One.*

Yes, there are several. The largest is the Sussex County Hospital, Brighton.

*Asthma.*

(189) Do you advise "Weidhaas" cure for asthma; if so, where can I get particulars?—*Southend.*

We cannot give medical advice. Should you not have called the method "treatment," and not "cure?" If your medical attendant knew of any certain cure, we feel sure that he would recommend it.

*Heart Disease.*

(190) Will you tell me of a home in North London where a poor woman suffering from heart disease could be received? Her sister could pay a very little.—*Mildmay.*

Apply to Woodside Home, Whetstone, N., and though not North London you might also find it worth while to write to St. Elizabeth's Home, 59 Mortimer Street, W.

*School Nurse.*

(191) I am a fully trained and experienced nurse. Can you tell me to whom I should apply for the post of school nurse?—*School.*

Write to the London and other County Councils. The office of the London Council is at Spring Gardens, S.W.

*Sponges.*

(192) A nurse in Paris would be glad to receive advice how to keep sponges from getting soft and nasty. She can only use them for bathing a patient a month or six weeks.—*C.M.B.*

We imagine that the nurse must either use soap with the sponge or that the sponges are bad. A sponge if not used to apply soap, and if rinsed in clean cold water after use, should last for many months. To cleanse sponges let them soak in boiling water with a handful of common salt for some time, and then squeeze out well, with clean water to finish.

*Rheumatism.*

(193) I am a trained nurse, and have been nursing in fever tents, since which I have had rheumatism so badly in my hands that at last I have had to give up my work. I have had medicine, but it has done me no good, and I fear that the doctor thinks the rheumatism is chronic. Can you tell me of a good specialist? I am recommended to try Bath or Buxton, but cannot afford it.—*Worried.*

Why not ask your medical attendant if he advises you to go to either Bath or Buxton? We feel sure that if as a nurse you write to the Secretary, the Royal Mineral Hospital, Bath, or the Devonshire Hospital and Buxton Bath Charity, Buxton, you will be received free of charge at one or the other.

**Handbooks for Nurses.**

| | Post Free. |
|---|---|
| "How to Become a Nurse: How and Where to Train." | 2s. 4d. |
| "Nursing: its Theory and Practice." (Lewis.) ... | 3s. 6d. |
| "Nurses' Pronouncing Dictionary of Medical Terms." | 2s. 6d. |
| "Complete Handbook of Midwifery." (Watson.) ... | 6s. 4d. |
| "Preparation for Operation in Private Houses." ... | 0s. 6d. |

Of all booksellers or of The Scientific Press, Limited, 28 & 29 Southampton Street, Strand, London, W.C.

### For Reading to the Sick.

PRAYER'S FULFILMENT.

THY prayer shall be fulfilled; but how?
His thoughts are not as thine,
While thou wouldst only weep and bow,
He saith, "Arise and shine!"
Thy thoughts were all of grief and night,
But His of boundless joy and light.
Thy father reigns supreme above:
The glory of His name
Is Grace and Wisdom, Truth and Love,
His will must be the same.
And thou hast asked all joys in one
In whispering forth, "Thy will be done."

*F. R. H.*

We are right to lay all our thoughts and wishes before God. We speak them as to a Father from whom we would hide nothing, even if we could. We do not fear to do so, if we indeed trust Him to guard and save us from our own wilfulness, and to grant only what He knows good for us. Right faith makes us thankful that He hears us, and also that He will not let us have our own foolish way. We should not dare to pray, if "Thy will be done" were not part of each petition. We show doubt of God, we mistake the meaning of prayer, if we think to bring God to our view of what is good for us; or are ready to withdraw our faith, unless He do our bidding in our way and when we like.—*Anon.*

The way of God which He would make us know is always the way of His will. The one business of life is to learn to do that will. We say it lightly in our prayers, "Thy will be done on earth, as it is in heaven." If our prayer is answered our whole life will be drawn into the divine way. We are to pray first for the hallowing of our Father's name. It is a great deal more important that we in our own life shall be interpreters of God, than that our burden shall be lifted away, our business prospered, our sorrows comforted. Next we are to pray for the coming of our Father's kingdom. This desire should be dearer to our heart than anything that concerns our own comfort.

There are particular times, also, when we need to make the prayer for direction with special earnestness. There are times when every star seems to have gone out, and when clouds and darkness appear to have gathered about us, hiding every waymark, so that we cannot see any way out of the gloom and perplexity.

Indeed, we often need the divine guidance the most when we think we do not need it at all. On the other hand, it is often true that the experiences we dread, in which we seem to be left without help, when the darkness appears most dense about us and we cannot see the way, even a step, before us, are really fullest of God. We cry out then for deliverance, not knowing that it is God who is leading us into the shadows. It is when the sun goes down that we see the stars.—*Dr. J. Miller.*

I know not which to choose; whether to live
A little longer here, or to depart.
And yet
To live for Christ, to live to do His pleasure. . .
. . . Which shall I choose, living, to live to Christ;
Or dying, die to Him, which shall I choose?
Whichever of the twain shall to Thy glory be,
That, Lord, I pray Thou wilt appoint for me.

*H. H. Swinney.*

## Figures and Tables

**Figure f1:**